# Changes in cholesterol metabolism-related gene expression in peripheral blood mononuclear cells from Alzheimer patients

**DOI:** 10.1186/1476-511X-11-39

**Published:** 2012-03-14

**Authors:** Antonella Mandas, Claudia Abete, Paolo Francesco Putzu, Paolo la Colla, Sandra Dessì, Alessandra Pani

**Affiliations:** 1 Department of Internal Medicine, University of Cagliari, Cittadella Universitaria, 09042- Monserrato (CA) Italy; 2 Department of Biomedical Sciences, University of Cagliari, Cittadella Universitaria, 09042- Monserrato (CA) Italy

**Keywords:** Alzheimer disease, Blood cells, Cholesterol, Cholesterol esters, Amyloid precursor protein, Neutral lipids, Biomarker

## Abstract

**Background:**

Cholesterol homeostasis dysfunction has been reported to have role in the pathogenesis of Alzheimer disease (AD). Therefore, changes in cholesterol metabolism in blood components may help to develop new potential AD biomarkers. In this study changes in cholesterol metabolism-related gene expression genes were evaluated in peripheral blood mononuclear cells (PBMCs) from AD subjects, their first degree relatives (FDR) and two groups of age matched controls (C1 > 80 years, C2 < 60 years). The expression of three genes related to APP processing was also determined.

**Results:**

Results showed significantly different behavior (P = 0.000) in the expression of all analyzed genes among the 4 groups. An inverse correlation emerged between the age of controls and the propensity of their PBMCs to express selected genes. Moreover, when gene expression was evaluated in PBMCs from AD patients and compared with that of PBMCs from healthy subjects of the same age, LDL-R and APP mRNAs were most abundant in AD as compared C1 whereas SREBP-2 and particularly nCEH were present at much lower mRNA levels in AD-PBMCs. This study describes for the first time a differential expression profile of cholesterol and APP related genes in PBMCs from AD patients and their FDR.

**Conclusions:**

We suggest that the expressions of cholesterol homeostasis and APP processing related genes in PBMC could be proposed as possible biomarkers to evaluate AD risk. In addition, gene expression in PBMC could be also used for diagnosis and development of therapeutic strategies as well as for personalized prediction in clinical outcome of AD.

## Background

Alzheimer disease (AD) is a severe neurodegenerative disorder characterized by loss of memory and cognitive decline that at a cellular level, exhibits several histopathological markers including beta-amyloid (Aβ) plaques, formed after sequential cleavage by β and γ secretases of the amyloid precursor protein (APP), neurofibrillary tangles (NFTs) within neurons, and the loss of synaptic connections manifested as brain atrophy [1-3]. The prevalence of AD is expected to rise dramatically in the next few decades, thus, it is a great challenge to establish reliable surrogate markers to diagnose and monitor the progression of this devastating disease. However, the development of these biomarkers is complicated not only for the variability in clinical features and multiple molecular etiologies, but especially for the impossibility to make analyses on brain *in vivo*. During the last several decades, the knowledge pertaining to brain cellular cholesterol homeostasis has greatly increased and numerous studies have indicated that changes in intracerebral cholesterol levels are involved in the pathogenesis of AD and possibly other forms of neuropathologies [4]. It has been demonstrated that increased cholesterol levels stimulate the activity of the β-secretase (BACE-1) pathway, leading to an accumulation of Aβ peptides [5] On the other hand, it has been also reported that Aβ itself may affect cholesterol homeostasis by reducing intracellular free cholesterol (FC) levels [6]. In CHO cells and various neuron-like cells grown in culture, the decrease of cholesterol esters (CE) either by genetic inactivation of acylCoA:cholesterol-acyltransferase (ACAT-1), the intracellular enzyme that catalyzes CE synthesis, or by pharmacological inhibition of this enzyme, was associated with a decrease of Aβ secretion [7]. In addition, in an AD mouse model ACAT inhibitors CP-113,818 and CI 1011 substantially diminished amyloid plaque density [8,9]. Based on these studies, it has been suggested that the ratio between FC and total cholesterol (TC) is a primary regulator of the APP processing, and that ACAT may be considered as a drug target for therapeutic intervention against certain form(s) of AD [10]. In this regard, the ablation of ACAT-1 in AD-mice, was able to reduce more than 60% the full-length APP protein as well as its proteolytic fragments, and to ameliorate cognitive deficits [11]. Because of the extremely high ratio between FC and CE in the adult human brain, at the present it is difficult to understand how ACAT modulates APP processing in vivo, however, the aforementioned studies address cholesterol homeostasis as a central component of neurodegenerative cascades, which can influence several aspects of AD development. Thus, it seems that to prevent and treat neurodegenerative conditions, research needs to develop simple, inexpensive and rapid tools to measure several biomarkers involved in the modulation of cholesterol homeostasis in the brain. Unfortunately, the notion that cholesterol in the brain is insulated from changes in circulating cholesterol [12], limits the use of blood samples for cholesterol biomarker discovery in brain. An unbalance between FC and CE pools, similar to that observed in neuron-like cells grown in culture and in AD brain, however, was recently found by us in skin fibroblasts [13] as well as in peripheral blood mononuclear cells (PBMC) from AD patients and their first degree relative [14,15]. We suggested that PBMCs may be utilized as surrogate tissues to further investigate potentially harmful effects of cholesterol changes in tissues that are not otherwise easily accessible. In this study, we investigated changes in mRNA levels of the major proteins involved in: (1) cholesterol uptake: low density lipoprotein receptor, LDL-R; (2) cholesterol neosynthesis and regulation: hydroxy-methyl-glutaryl coenzyme A reductase, HMGCoA-R, and the sterol regulatory element binding protein-2, SREBP-2, a transcription factor regarded as the main regulator of cholesterol homeostasis; (3) cholesterol trafficking: caveolin-1 (Cav-1) and ATP binding cassette-A, ABCA-1; (4) cholesterol ester cycle: ACAT-1 and neutral cholesterol ester hydrolase, nCEH; (5) Aβ production: APP and BACE-1 and (6) Aβ degradation: neprilysin, in peripheral blood mononuclear cells (PBMCs) isolated from AD patients, and from their first degree relative (FDR-AD). As controls we utilized PBMCs from apparently healthy subjects aged > 75 years (controls 1) and < 60 years (controls 2) were employed as the normal counterparts. It was our aim the identification of differentially expressed genes and/or distinctive patterns of gene expression which may help to better understanding AD pathophysiology and to develop specific diagnosis and effective therapy.

## Results

### Measurement of neutral lipids in PBMCs of AD and FDR-AD subjects

**Figure 1:**
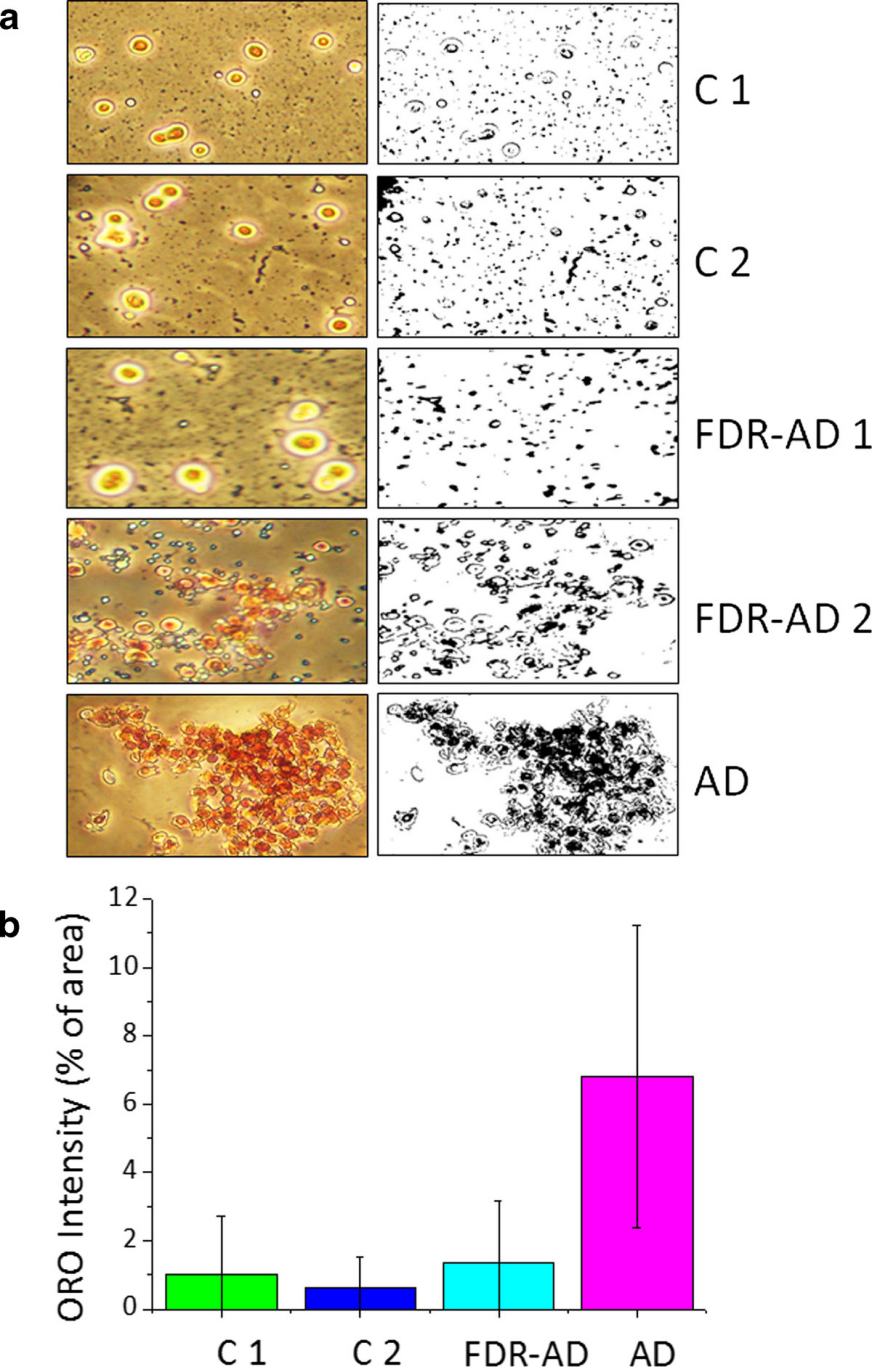
**ORO staining of cytoplasmic neutral lipids**. Freshly isolated PBMCs were stained with ORO to demonstrate neutral lipids and counterstained with haematoxylin for nuclei. Cells were then examined by light microscopy and two different fields per sample were imaged. Red ORO intensity was measured in these two fields using NIH Image J software. Panel (a) shows representative images of ORO stained (on the left) and their corresponding ROI (on the right). Panel (b) shows quantization of red intensity expressed as the percentage of pixel intensity ± SD⁄ total area of each image. Analysis of variance (ANOVA) shows significant statistically differences among the four groups (P = 0.000).

We began to determine neutral lipids in PBMCs separated from blood samples of 50 subjects: 13 subjects apparently healthy, lacking of cognitive deficit, aged between 81-87 years (C1), 12 subjects diagnosed with late-onset probable AD, aged between 70-89 years (AD), 12 subjects apparently healthy, aged between 25-57 years (C2) and 13 FDR of AD subjects (FDR-AD) aged between 40-63 years. Total neutral lipids were determined by staining freshly isolated PBMCs with Oil-red-O (ORO), a lysochrome (fat soluble dye) widely used for demonstrating the presence of neutral lipids (mostly CE) which appear as red-stained cytoplasmic droplets. ORO staining evidenced a consistent accumulation of neutral lipids in cytoplasm of all AD-PBMCs compared to C1 (Figure 1a). Two different pictures have indeed been observed in FDR-AD (Figure 1a). Six out of 13 of them (46%) exhibited neutral lipid pattern similar to their AD affected relatives, while in 7 subjects, neutral lipids were absent or very low resembling that of C2. Quantization of red intensity in each image was also determined using the public domain program Image J 1.42 and selecting a region of interest (ROI). The comparison of these pictures, by analysis of variance (ANOVA), showed a significant difference (P = 0.000) among the groups (Figure 1b). These results confirmed that alterations of lipid metabolism in peripheral cells, i.e. PBMCs, are associated with AD. It remains to be established whether they are merely due to a defect in ACAT activity, or rather they reflect differences in cholesterol pools or in cellular cholesterol distribution.

### mRNA levels of genes associated with cholesterol homeostasis and APP processing in ex vivo PBMCs

**Figure 2:**
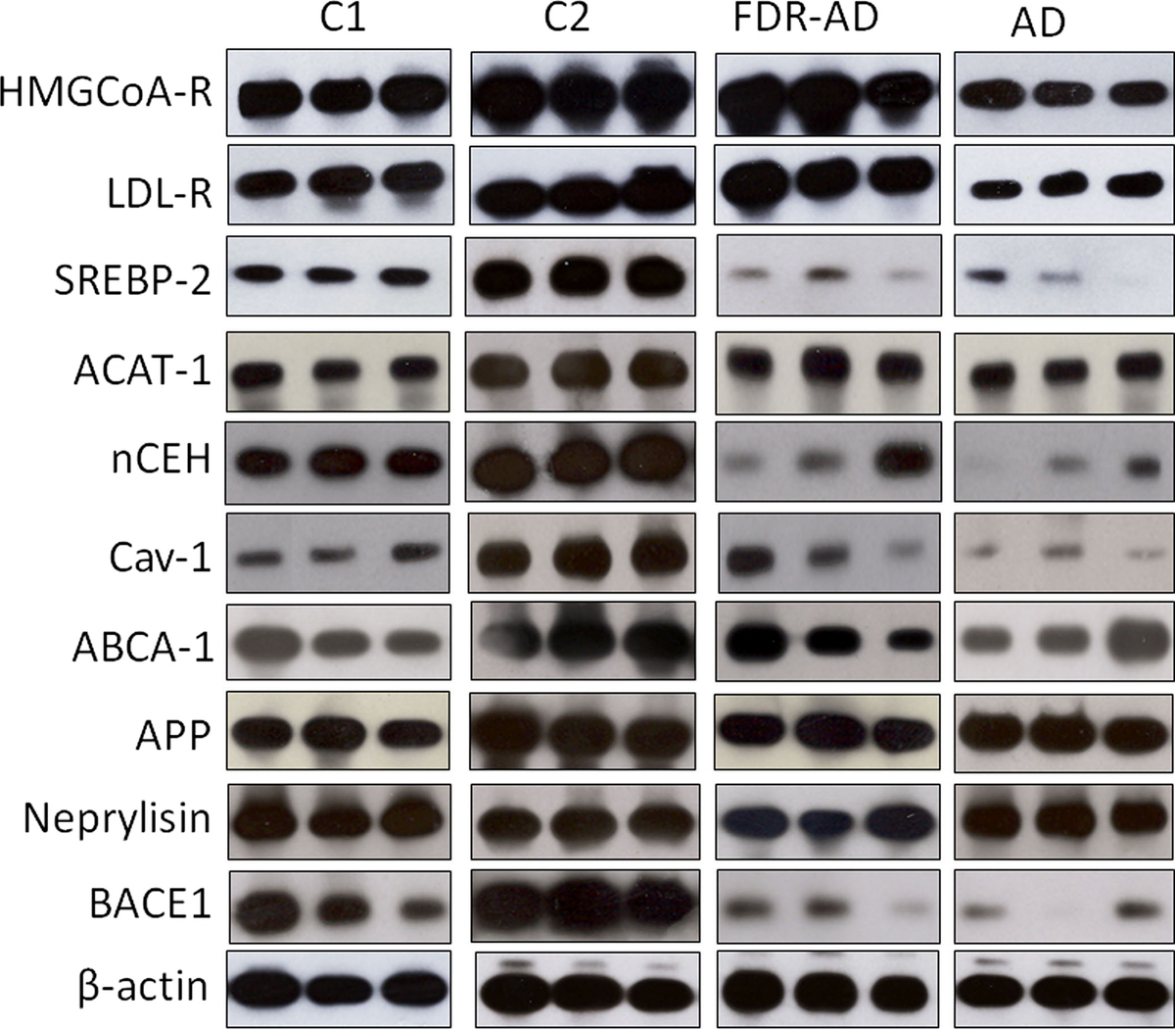
**Representative blots of mRNA levels of cholesterol metabolism and APP processing-related genes**. Total mRNA was extracted from PBMCs of the selected groups. mRNA levels of indicated genes were then determined by RT-PCR using appropriate primer sets. Specific bands were detected after addition of a chemiluminescent substrate.

**Figure 3:**
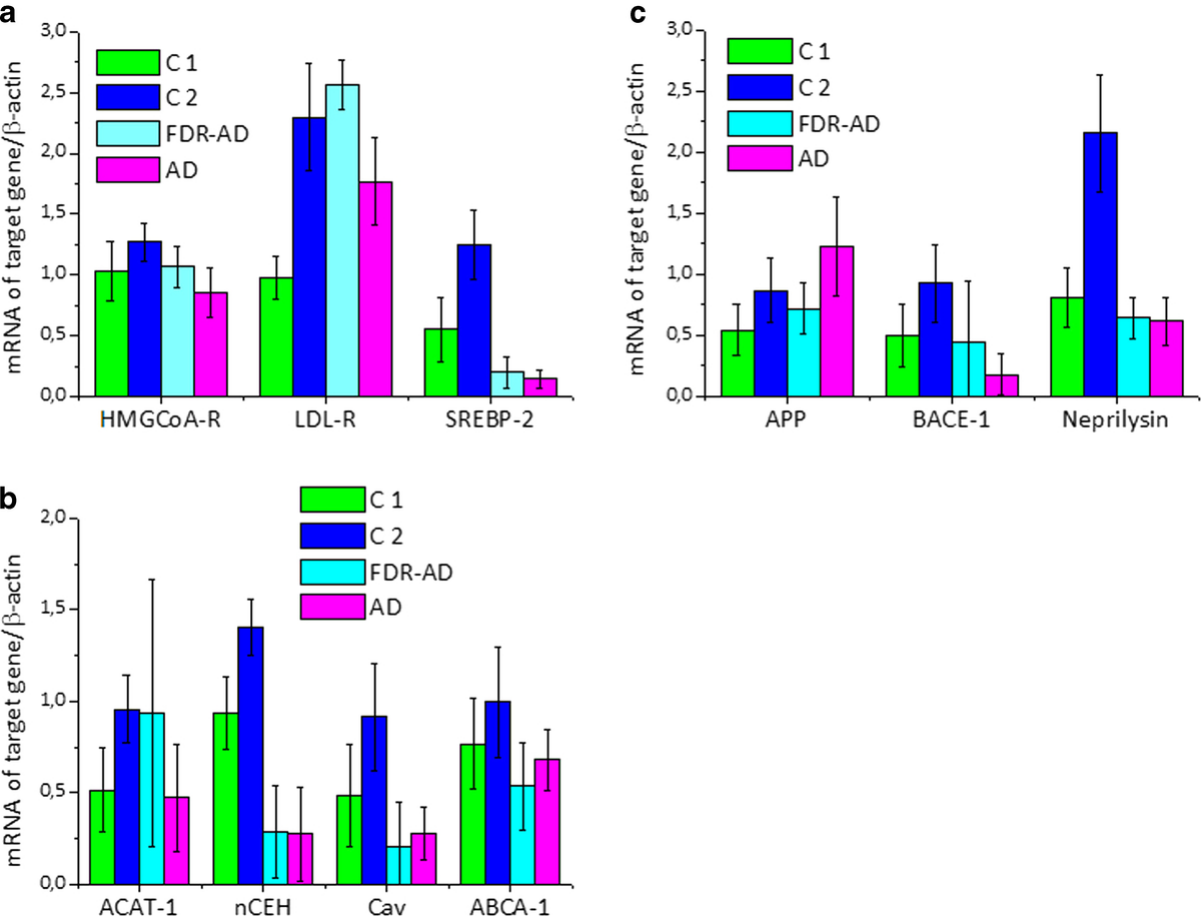
**Densitometric analysis of mRNA levels of target genes normalized for the endogenous β-actin**. mRNA levels were quantified by using NIH Image 1.63 program (Scion Image). Data values are represented as mean ± SD for each group. *Statistical analysis performed by using the one-way ANOVA test showed highly significant differences among the groups (P = 0.000 for all genes, with the exception of ACAT-1 for whom the significance was P = 0.006).

**Figure 4:**
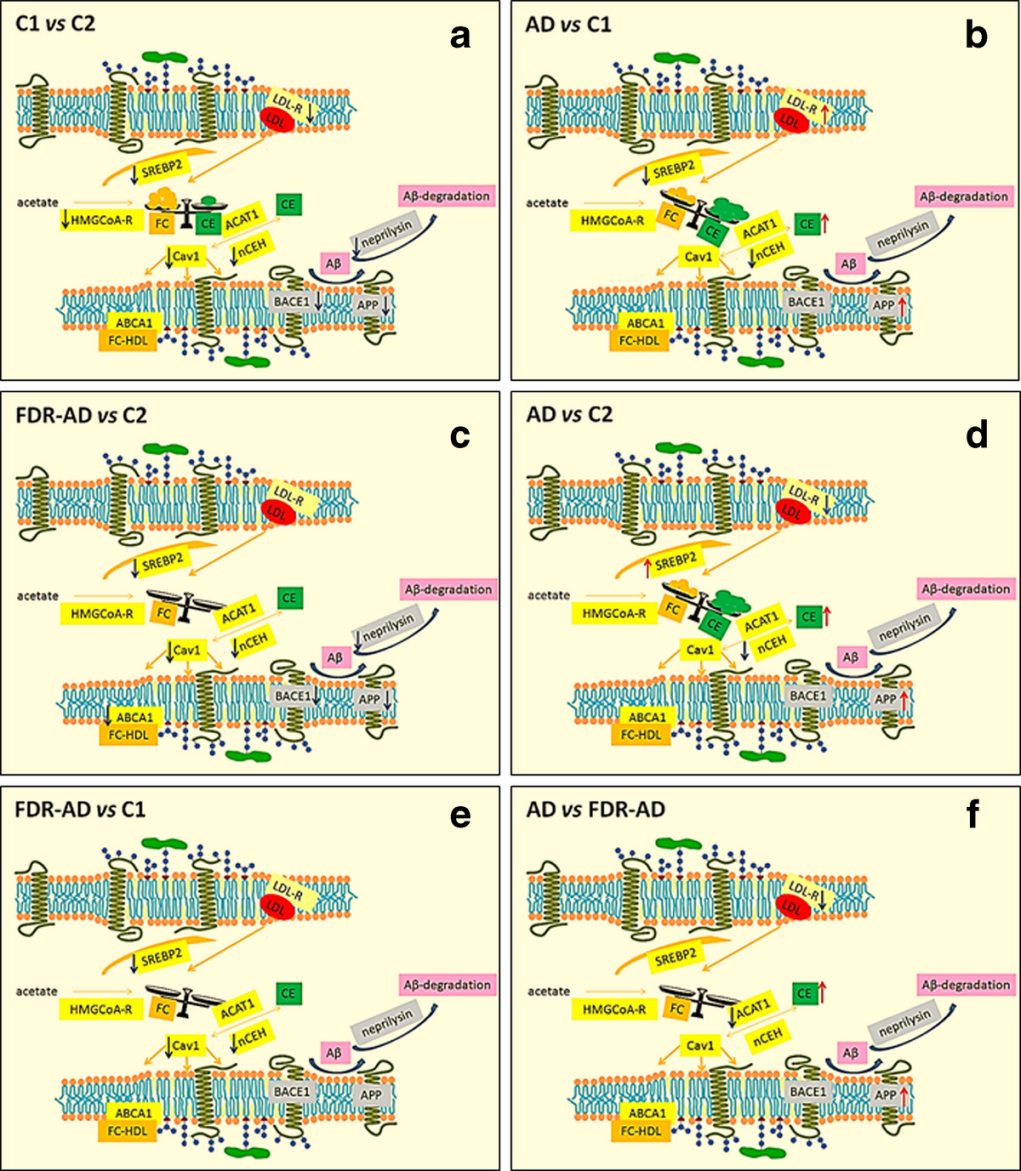
**Schematic representation of the data collected from the mRNA quantifications**. The schemes represent the expression level, cellular compartment and metabolic network position of genes linked to cholesterol homeostasis and to APP processing. The arrows (↑↓) denote the significant (P < 0.05) differences (increase or decrease, respectively) between the two indicated groups, obtained by applying a Bonferroni's multiple comparison test following ANOVA.

To gain insights into the possibility that gene expression data in blood cells may enable the identification of potential new biomarkers for brain diseases including AD, we next determined the mRNA levels of a number of genes associated with cholesterol homeostasis and APP processing in ex vivo PBMCs from the 4 selected groups (Figure 2). Analysis of variance (ANOVA) showed significantly different behavior (P = 0.000) in the expression of all analyzed genes between the groups (Figure 3). Table 1 shows association between age and genes involved in cholesterol and APP metabolism in both controls (C1 + C2), and AD + FDR-AD, as measured by the Pearson correlation coefficient. A significant inverse correlation emerged between the age of healthy controls and the propensity of their PBMCs to express selected genes. With the exception of ACAT-1 and ABCA-1, the mRNA levels of all other genes examined were decreased significantly (p < 0.05) in PBMCs of old subjects (C1) when compared to that of middle-aged volunteers (C2) (Figure 4a). However, when results obtained from AD patients were compared to that from C2 (AD vs C2) (Figure 4c), the accumulation of CE was associated with an increased expression of SREBP-2 and APP and with a decrease of ABCA-1 and ACAT-1. In addition, LDL-R and APP mRNAs were most abundant (p < 0.05) in AD compared to C1 (Figure 4b.) whereas SREBP-2 and particularly nCEH were present at much lower mRNA levels in AD PBMCs. No significant changes in the expression of the other genes were observed between AD and C1 groups (Figure 4b). A partially different scenario was observed when FDR-AD was compared to C1, C2 and AD groups (Figures 4e, d and 4f). Compared to C2, FDR-AD PBMCs exhibited lower expression of SREBP-2, Cav-1, nCEH, ABCA-1 as well as BACE-1, APP and neprylisin (Figure 4d), a pattern of gene expression similar to C1 group with the exception of SREBP-2, nCEH and Cav-1 mRNA levels that were significantly (p < 0.05) decreased in FDR-AD compared to C1 (Figure 4e). Compared to AD, FDR-AD subjects have lower CEs levels and lower mRNA APP levels; but higher mRNA levels of ACAT-1 (Figure 4f).

**Table 1:** Pearson's correlation coefficient (age vs mRNA levels of selected genes)

	C1 + C2 (n.25)	AD + FDR-AD (n.25)
Neprilysin	**r = -0,8992**	r = -0,2233

	**p = 0,000**	p = 0,283

ABCA-1	r = -0,3503	r = 0,2457

	p = 0,086	p = 0,236

ACAT-1	**r = -0,7171**	r = -03421

	**p = 0,000**	p = 0,094

APP	**r = -0,5171**	**r = 0,4477**

	**p = 0,008**	**p = 0,025**

BACE-1	**r = -0,5849**	r = -0,3629

	**p = 0,002**	p = 0,075

Cav-1	**r = -0,6192**	r = 0,0718

	**p = 0,001**	p = 0,733

HMGCoA-R	**r = -0,4843**	**r = -0,4748**

	**p = 0,014**	**p = 0,016**

LDL-R	**r = -0,8361**	**r = -0,6829**

	**p = 0,000**	**p = 0,000**

nCEH	**r = -0,7544**	r = -0,0151

	**p = 0,000**	p = 0,943

SREBP-2	**r = -0,7633**	r = -0,2629

	**p = 0,000**	p = 0,204

Significant statistical differences were indicated in bold

## Discussion

Histochemical and immunohistochemical detection of Aβ in postmortem brain tissue still remains the most accurate and definitive means by which AD is diagnosed. Starting from the notion that AD neurons inappropriately enter the cell cycle without to complete it, recent studies have proposed neuronal dysfunction and death secondary cell cycle abnormalities, as sources of potential AD biomarkers [16]. Similarly, oxidative damage to various proteins, nucleic acids and other compounds found early in AD neurons, probably arising from mitochondrial abnormalities, has been also proposed as a biomarker of AD risk [1,17,18]. In addition, more recently, scientists from Pittsburgh University, have developed a radio fluorinated positron emission tomography (PET) probe, 2-(1-{6-[(2-[F 18]fluoroethyl)(methyl)amino]-2-naphthyl}ethylidene)malononitrile ([F-18]FDDNP), having an high binding affinity for amyloid and amyloid-like structures, for detection of in vivo brain imaging that allows to distinguish between AD patients and healthy controls [19]. The rational was that AD begins with abnormal processing of APP, which leads to deposits of β- amyloid (Aβ) in the neocortical and subcortical regions of the brain [3]. Although undoubtedly suggestive all the above strategies have restricted applicability and are not suitable for routine use, and hence, the development of techniques non-invasive, simple, cheap, using easily accessible samples, remains urgently needed for early diagnosis of AD. A large body of evidence now suggests that APP metabolism is affected by alteration of the lipid microenvironment, however, relative few studies in this matter have been performed in humans where an important barrier to identify molecular biomarkers was the inaccessibility to brain samples [20]. Thus, keeping these questions in mind it would be useful to find a noninvasive source of nucleic acids to explore the role of genes involved in cholesterol homeostasis in the context of human APP processing. An important component in AD and cholesterol metabolism research concerns the role of the isoforms (ε2,ε3,ε4) of apolipoprotein E (ApoE), a plasma protein involved in the transport of cholesterol in the central nervous system. The presence of ApoE ε4 isoform, determined by using DNA from PBMC, has been repeatedly proposed as a risk factor for AD [21,22]. However, the use of ApoE genotype as a diagnostic test in symptom-free individuals has limited applicability since it cannot predict with certainty when, or if, an individual with ApoE ε4/4 will get AD. Peripheral tissues suitable for providing useful molecular markers for diagnosis of AD include skin fibroblasts, platelets, lymphocytes [23]. A previous our study showing a significant higher concentration of neutral lipids in PBMCs from AD patients and in some of their FDR[15] suggested that this kind of sample could be applied to perform studies on lipid-related molecular targets involved in AD. In this sense, the current experimental study has specifically utilized PBMCs collected by simple blood samples to assess the expression of a large number of genes correlated to cholesterol homeostasis and APP processing in AD patients and their younger FDR. Our results showed that, during physiological aging, PBMCs, despite of a general decline of cholesterol turnover; retain the molecular mechanisms responsible for the regulation of intracellular cholesterol levels. These results are in agreement with the notion that cholesterol turnover relatively high in the developing brain, strongly declines to a very low level in the aging state [24]. In addition, consistent with the notion that at the molecular level, intracellular cholesterol regulates APP processing and Aβ production [25], the age-related decline in cholesterol turnover was closely associated with an age-related decrease in the expression of genes normally involved in APP processing. However, when gene expression was evaluated in PBMCs from AD patients and compared with that of PBMCs from healthy subjects of the same age (C1), LDL-R and APP mRNAs were most abundant in AD compared to C1 whereas SREBP-2 and particularly nCEH were present at much lower RNA levels in AD-PBMCs. These results suggested that at least some of physiologic cholesterol homeostatic mechanisms are impaired in AD patients and that these altered mechanisms are correlated with an increased gene expression of APP. In addition, they provide indirect evidence that besides ACAT1, nCEH, the enzyme responsible for hydrolysis of CE, plays a major role in neutral lipids accumulation observed in AD-PBMCs. The elevated steady-state mRNA levels of APP observed in AD-PBMCs may be responsible for the increased Aβ production observed in vitro and in vivo in AD models [26,27]. It remains uncertain whether and how BACE1 is associated with aging or neurodegenerative disease since under our experimental conditions BACE1 mRNA levels significantly decreased during physiological aging and beside the increase in the expression of APP, they remain unchanged in AD-PBMCs. In this study, we also showed that in AD-FDR-PBMCs, several cholesterol related genes are regulated in the same direction of those involved in APP processing: lower expression of SREBP-2, Cav-1, nCEH, ABCA1 as well as that of BACE1, APP and neprylisin were observed in AD-FDR compared to C1. Compared to AD, mRNA expression of ACAT1 in AD-FDR appears to negatively correlate with APP mRNA levels, a pattern of gene expression similar to C1 group with the exception of SREBP2, nCEH and Cav 1 mRNA levels that were significantly decreased in AD-FDR compared to C1.

## Conclusions

The current study demonstrates that the use of circulating mononuclear cells in the search of transcriptional biomarkers in the context of neurodegenerative disorders is feasible. Moreover, it reported for the first time a differential expression profile of cholesterol and APP related genes in PBMCs from AD patients and their FDR. This is of particular relevance given the current view that genetic factors play an important role in many cases of sporadic AD [28]. Even though these findings should be corroborated in future studies, the expressions of cholesterol homeostasis and APP processing related genes in PBMC could be proposed as possible biomarkers to evaluate AD risk. In addition, gene expression in PBMC could be also used for diagnosis and development of therapeutic strategies as well as for personalized prediction in clinical outcome of AD.

## Methods

### Subjects

Blood samples were obtained from 50 subjects randomly selected from a list of 400 subjects that were enrolled in a pre-existing study [14]. They were divided into four groups according to age: C1, old normal subjects (mean age 83.8 ± 1.6 years); C2, middle-age normal subjects (mean age 48.6 ± 10.5 years); FDR-AD, first degree relatives of AD patients without clinical symptoms (51.40 ± 10.3 years); AD, patients with clinical evidence of neuropathy (80.1 ± 5.9 years). Informed written consent was obtained from all patients and healthy controls before initiating the study, according to the policies of the hospital's Institutional Review Boards. Individuals included in this study were enrolled at the Geriatric Units of Aziende Ospedaliero-Universitarie and ASL 8 of Cagliari (Italy). Control subjects were healthy volunteers and none of them was a relative or caregiver of the AD patients or seeking medical care for any reason. Subjects with neoplastic or hematological disorders, recent infections or surgery, severe hepatic or renal failure, myocardial infarction or cranial trauma in the previous 6 months, or who had received statins, antineoplastic, corticosteroid, or immunosuppressive drug treatments were not included in the study. The diagnosis of probable AD was made according to the criteria developed by National Institute of Neurological and Communicative Disorders and Stroke (NINCDS) and the Alzheimer's Disease and Related Disorders Association (ADRDA). For AD, routine clinical and laboratory evaluation was performed to exclude other causes of cognitive impairment and all subjects received neuropsychological tests, such as the Mini-Mental State Examination (MMSE). To assess the severity of the cognitive impairment in AD patients, the Reisberg Global Deterioration Scale (GDS) was also used. Abnormal GDS levels start from level 3 and maximal deterioration grade corresponds to level 7. Patient evaluation included medical history, physical and neurological examinations, laboratory blood tests to rule out metabolic causes of dementia (thyroid hormones, vitamin B12, and erythrocyte sedimentation rate), and brain neuroimaging (computed tomography and/or magnetic resonance).

### Cell types and culture conditions

PBMCs were obtained by centrifuging blood samples at 600 g for 15 min. After centrifugation, plasma was removed and the buffy coat was collected, and PBMCs separated by Ficoll-Hypaque density gradient. Cells were then suspended (1 × 10^6 ^cells ⁄ ml) in RPMI-1640 and processed immediately (ex vivo) for lipid staining or frozen at -80°C for mRNA.

### Neutral lipid staining

Cells cultured as described above were treated with isopropyl alcohol (60%), washed, stained in oil red O (ORO) for NL and counterstained with Mayer's haematoxylin. Stained cells were examined by light microscopy. Cytoplasmic red-stain intensity indicating neutral lipid accumulation was quantified using Image J software (National Institutes of Health, United States). ORO intensity was calculated as percentage of positively stained area (pixels/μm^2^) obtained by manually selecting one region of interest (ROI).

### Reverse-transcriptase polymerase chain reaction (RT-PCR) and Southern blotting

mRNA levels for low density lipoprotein receptor (LDLR), hydroxy-methyl-glutaryl coenzyme A reductase (HMGCoA-R), sterol regulatory element-binding protein-2 (SREBP-2), ATP-binding cassette A (ABCA1), acylCoA-cholesterol acyltransferase (ACAT-1), neutral cholesterol ester hydrolase (nCEH), and caveolin-1 (cav-1), were evaluated by reverse transcription polymerase chain reaction (RT-PCR) using appropriate primer sets. We chose to utilize this technique since our experience indicates that it can be set up rapidly in a clinical laboratory, and provide sensitive and specific results at relatively low cost. Real-time PCR, by using fluorescently labeled oligonucleotide probes, allows to detect and quantitate a specific PCR product in real time. However, it is difficult to design and requires more expensive laboratory equipment. Total RNA was extracted from approximately 10^6 ^cells using TRIZOL reagent (Invitrogen Corporation, Carlsbad, CA, USA). Equal amounts of total RNA (1 μg) were reverse transcribed into cDNA using the random hexamer method and amplified by PCR in presence of specific primers, according to the manufacturer's instructions (GeneAmp RNA PCR Kit; Perkin-Elmer Cetus, Norwalk, CT, USA). Amplicons were labelled during PCR, with digoxigenin-11-dUTP (Roche Applied Science, Mannheim, Germany), immunodetected with anti-digoxigenin antibodies conjugated to alkaline phosphatase (Roche Applied Science) and visualized with chemiluminescent substrate CSPD. Intensity of autoradiographic bands was measured after exposure to X-ray film using Kodak Digital Science Band Scanner Image Analysis System (Kodak, Rochester, NY, USA). Specific bands were detected and analyzed by NIH Image 1.63 program (Scion Image, Frederick, MD, USA). Amounts of PCR products for each target mRNA was normalized by using β-actin as housekeeping gene.

### Statistical analysis

Data are reported as mean ± standard deviation (SD). Statistical calculations were performed using statistical analysis software Origin 8.0 version (Microcal Inc, Northampton, MA, USA). Statistical comparisons between the four groups were made by using a one-way ANOVA and where appropriate a post hoc Bonferroni test and Pearson correlation coefficient. A probability of p < 0.05 was considered statistically significant.

## Abbreviations

AD: Alzheimer disease; Aβ: beta-amyloid; APP: Amyloid precursor protein; FC: Free cholesterol; CE: Cholesterol esters; ACAT-1: AcylCoA:cholesterol-acyltransferase; PBMC: Peripheral blood mononuclear cells; LDL-R: Low density lipoprotein receptor; HMGCoA-R: Hydroxy-methyl-glutaryl coenzyme A reductase; SREBP-2: Sterol regulatory element binding protein-2; Cav-1: Caveolin-1; ABCA-1: ATP binding cassette-A; NCEH: Neutral cholesterol ester hydrolase; FDR: First degree relative; ORO: Oil-red-O

## Competing interests

The authors declare that they have no competing interests.

## Authors' contributions

AM, PFP, PLC, SD and AP designed the study; CA performed the experiments; AM and SD wrote the paper. All authors read and approved the final manuscript.

## References

[B1] Reddy PH Beal MF Amyloid beta, mitochondrial dysfunction and synaptic damage: implications for cognitive decline in aging and Alzheimer's disease Trends Mol Med 2008 14 45 53 10.1016/j.molmed.2007.12.002 18218341 PMC3107703

[B2] Small DH Network dysfunction in Alzheimer's disease: does synaptic scaling drive disease progression? Trends Mol Med 2008 14 103 108 10.1016/j.molmed.2007.12.006 18262842

[B3] Zhang YW Xu H Molecular and cellular mechanisms for Alzheimer's disease: understanding APP metabolism Curr Mol Med 2007 7 687 696 10.2174/156652407782564462 18045146

[B4] Puglielli L Tanzi RE Kovacs DM Alzheimer's disease: the cholesterol connection Nat Neurosci 2003 6 345 351 10.1038/nn0403-345 12658281

[B5] Ghribi O Larsen B Schrag M Herman MM High cholesterol content in neurons increases BACE, β-amyloid, and phosphorylated tau levels in rabbit hippocampus Exper Neurol 2006 200 460 467 10.1016/j.expneurol.2006.03.019 16696972

[B6] Huttunen HJ Puglielli L Ellis BC MacKenzie Ingano LA Kovacs DM Novel N-terminal cleavage of APP precludes Abeta generation in ACAT-defective AC29 cells J Mol Neurosci 2009 37 6 15 10.1007/s12031-008-9088-0 18618086 PMC2721794

[B7] Puglielli L Konopka G Pack-Chung E Ingano LA Berezovska O Hyman BT Acyl-coenzyme A: cholesterol acyltransferase modulates the generation of the amyloid beta-peptide Nat Cell Biol 2001 3 905 912 10.1038/ncb1001-905 11584272

[B8] Hutter-Paier B Huttunen HJ Puglielli L Eckman CB Kim DY Hofmeister A The ACAT inhibitor CP-113,818 markedly reduces amyloid pathology in a mouse model of Alzheimer's disease Neuron 2004 44 227 238 10.1016/j.neuron.2004.08.043 15473963

[B9] Puglielli L Ellis BC Ingano LA Kovacs DM Role of acyl-coenzyme a: cholesterol acyltransferase activity in the processing of the amyloid precursor protein J Mol Neurosci 2004 24 93 96 10.1385/JMN:24:1:093 15314256

[B10] Huttunen HJ Kovacs DM ACAT as a drug target for Alzheimer's disease Neurodegener Dis 2008 5 212 214 10.1159/000113705 18322393 PMC2720799

[B11] Bryleva EY Rogers MA Chang CC Buen F Harris BT Rousselet E ACAT1 gene ablation increases 24(S)-hydroxycholesterol content in the brain and ameliorates amyloid pathology in mice with AD Proc Natl Acad Sci USA 2010 107 3081 3086 10.1073/pnas.0913828107 20133765 PMC2840286

[B12] Dietschy JM Turley SD Cholesterol metabolism in the brain Curr Opin Lipidol 2001 12 105 112 10.1097/00041433-200104000-00003 11264981

[B13] Pani A Dessì S Diaz G La Colla P Abete C Mulas C Altered cholesterol ester cycle in skin fibroblasts from patients with Alzheimer's disease J Alzheimers Dis 2009 18 829 841 19749436 10.3233/JAD-2009-1193

[B14] Pani A Mandas A Diaz G Abete C Cocco PL Angius F Accumulation of neutral lipids in peripheral blood mononuclear cells as a distinctive trait of Alzheimer patients and asymptomatic subjects at risk of disease BMC Med 2009 7 66 10.1186/1741-7015-7-66 19883495 PMC2777188

[B15] Pani A Mandas A Dessì S Cholesterol, Alzheimer's disease, prion disorders: a ménage à trois? Curr Drug Targets 2010 11 1018 1031 10.2174/138945010791591386 20450474

[B16] McShea A Lee HG Petersen RB Casadesus G Vincent I Linford NJ Neuronal cell cycle re-entry mediates Alzheimer disease-type changes Biochim Biophys Acta 2007 1772 467 472 17095196 10.1016/j.bbadis.2006.09.010

[B17] Moreira PI Santos MS Oliveira CR Shenk JC Nunomura A Smith MA Alzheimer disease and the role of free radicals in the pathogenesis of the disease CNS Neurol Disord Drug Targets 2008 7 3 10 10.2174/187152708783885156 18289026

[B18] Wang X Su B Zheng L Perry G Smith MA Zhu X The role of abnormal mitochondrial dynamics in the pathogenesis of Alzheimer's disease J Neurochem 2009 109 153 159 19393022 10.1111/j.1471-4159.2009.05867.x PMC2720030

[B19] Kepe V Huang SC Small GW Satyamurthy N Barrio JR Visualizing pathology deposits in the living brain of patients with Alzheimer's disease Methods Enzymol 2006 412 144 160 17046657 10.1016/S0076-6879(06)12010-8

[B20] Beela AJ Sakakuraa M Barretta PJ Sanders CR Direct binding of cholesterol to the amyloid precursor protein: An important interaction in lipid-Alzheimer's disease relationships? BBA - Mol Cell Biol Lipid 2010 1801 975 982 10.1016/j.bbalip.2010.03.008 PMC2886191 20304095

[B21] Kim J Basak JM Holtzman DM The Role of Apolipoprotein E in Alzheimer's Disease Neuron 2009 63 287 303 10.1016/j.neuron.2009.06.026 19679070 PMC3044446

[B22] Gustaw-Rothenberg K Lerner A Bonda DJ Lee HG Zhu X Perry G Biomarkers in Alzheimer's disease: past, present and future Biomark Med 2010 4 15 26 10.2217/bmm.09.86 20387301 PMC2855161

[B23] Gasparini L Racchi M Binetti G Trabucchi M Solerte SB Alkon D Peripheral markers in testing pathophysiological hypotheses and diagnosing Alzheimer's disease FASEB J 1998 12 17 34 9438407 10.1096/fasebj.12.1.17

[B24] Dietschy JM Turley SD Cholesterol metabolism in the central nervous system during early development and in the mature animal J Lipid Res 2004 45 1375 1397 10.1194/jlr.R400004-JLR200 15254070

[B25] Paz Marzolo M Bu G Lipoprotein receptors and cholesterol in APP trafficking and proteolytic processing, implications for Alzheimer's disease Semin Cell Dev Biol 2009 20 191 200 10.1016/j.semcdb.2008.10.005 19041409 PMC2691858

[B26] Naslund J Haroutunian V Mohs R Davis KL Davies P Greengard P Correlation between elevated levels of amyloid beta-peptide in the brain and cognitive decline JAMA 2000 283 1571 1577 10.1001/jama.283.12.1571 10735393

[B27] Oddo S Caccamo A Shepherd JD Murphy MP Golde TE Kayed R Triple-transgenic model of Alzheimer's disease with plaques and tangles: intracellular Abeta and synaptic dysfunction Neuron 2003 39 409 421 10.1016/S0896-6273(03)00434-3 12895417

[B28] Xiong L Gaspar C Rouleau GA Genetics of Alzheimer's Disease and Research Frontiers in Dementia Ger Aging 2005 8 31 35

